# Molecular epidemiological study of adenovirus infecting western lowland gorillas and humans in and around Moukalaba-Doudou National Park (Gabon)

**DOI:** 10.1007/s11262-016-1360-8

**Published:** 2016-06-11

**Authors:** Chimène Nze Nkogue, Masayuki Horie, Shiho Fujita, Michiko Ogino, Yuki Kobayashi, Keijiro Mizukami, Tatsunori Masatani, Sayeh Ezzikouri, Aya Matsuu, Tetsuya Mizutani, Makoto Ozawa, Osamu Yamato, Alfred Ngomanda, Juichi Yamagiwa, Kyoko Tsukiyama-Kohara

**Affiliations:** 1 Department of Pathological and Preventive Veterinary Science, The United Graduate School of Veterinary Science, Yamaguchi University, Yamaguchi, Japan; 2 Institut de Recherche en Ecologie Tropicale (IRET), Centre National de Recherche Scientifique et Technologique (CENAREST), Libreville, Gabon; 3 Transboundary Animal Diseases Research Centre, Joint Faculty of Veterinary Medicine, Kagoshima University, Kagoshima, Japan; 4 Department of Behavioral Physiology and Ecology, Joint Faculty of Veterinary Medicine, Kagoshima University, Kagoshima, Japan; 5 Faculty of Agriculture, Yamaguchi University, Yamaguchi, Japan; 6 College of Bioresource Sciences, Nihon University, Fujisawa, Japan; 7 Laboratory of Clinical Pathology, Joint Faculty of Veterinary Medicine, Kagoshima University, Kagoshima, Japan; 8 Virology Unit, Viral Hepatitis Laboratory, Institut Pasteur du Maroc, Casablanca, Morocco; 9 Research and Education Center for Prevention of Global Infectious Diseases of Animals, Tokyo University of Agriculture and Technology, Fuchu, Japan; 10 Kyoto University, Kyoto, Japan

**Keywords:** Adenoviridae, Gorillas, Gabon, Phylogeny

## Abstract

Adenoviruses are widespread in human population as well as in great apes, although the data about the naturally occurring adenovirus infections remain rare. We conducted the surveillance of adenovirus infection in wild western lowland gorillas in Moukalaba-Doudou National Park (Gabon), in order to investigate naturally occurring adenovirus in target gorillas and tested specifically a possible zoonotic transmission with local people inhabiting the vicinity of the park. Fecal samples were collected from western lowland gorillas and humans, and analyzed by PCR. We detected adenoviral genes in samples from both gorillas and the local people living around the national park, respectively: the overall prevalence rates of adenovirus were 24.1 and 35.0 % in gorillas and humans, respectively. Sequencing revealed that the adenoviruses detected in the gorillas were members of *Human mastadenovirus B* (HAdV-B), HAdV-C, or HAdV-E, and those in the humans belonged to HAdV-C or HAdV-D. Although HAdV-C members were detected in both gorillas and humans, phylogenetic analysis revealed that the virus detected in gorillas are genetically distinct from those detected in humans. The HAdV-C constitutes a single host lineage which is compatible with the host-pathogen divergence. However, HAdV-B and HAdV-E are constituted by multiple host lineages. Moreover, there is no evidence of zoonotic transmission thus far. Since the gorilla-to-human transmission of adenovirus has been shown before, the current monitoring should be continued in a broader scale for getting more insights in the natural history of naturally occurring adenoviruses and for the safe management of gorillas’ populations.

**Electronic supplementary material:**

The online version of this article (doi:10.1007/s11262-016-1360-8) contains supplementary material, which is available to authorized users.

## Introduction

Adenoviruses (AdVs) are non-enveloped icosahedral double-stranded DNA viruses. They belong to the family of *Adenoviridae*, which is divided into five genera: *Mastadenovirus*, *Atadenovirus*, *Aviadenovirus*, *Siadenovirus*, and *Ichtadenovirus*. Members of species belonging to genera *Mastadenovirus* and *Atadenovirus* are known to infect mammalian hosts [1, 2]. Mastadenoviruses infecting primates encompass seven *Human mastadenovirus* species (HAdV-A to G), the accepted species *Simian mastadenovirus A* and candidate species SAdV-B to G, and further not yet classified mastadenoviruses [2–4]. That classification into species is based on hemagglutination features, DNA (deoxyribonucleic acid) homology, and genomic organization [5]. There are currently over 60 HAdV types with HAdV-D containing the most members [5].

Adenoviruses were first isolated from humans and identified as the causative agent of epidemic febrile respiratory disease among military recruits in the 1950s [6, 7]. It is estimated that more than 90 % of the human population is seropositive for one or more serotypes of adenoviruses [8, 9]. The molecular biology of human-derived adenoviruses has been characterized extensively for species HAdV-C, for which human adenovirus 2 (HAdV-2) and HAdV-5 serve as prototypes [10]. Adenoviruses cause a variety of non-lethal infectious diseases in humans, and lethal disseminated adenovirus infection occurs in immunosuppressed patients [10].

The first description of a simian adenovirus in the literature was of a chimpanzee AdV [11], today known as SAdV-21 within the species *H. mastadenovirus B*. Later, when investigating chimpanzees suffering from kuru, four novel ape AdVs were discovered [12]. Ape AdVs have been detected or isolated from African apes including chimpanzees, bonobos, and gorillas [13–18]. Gorilla adenoviruses have been proposed to be members of HAdV-B, C, E, and F [13–18]. A recent report confirmed that the species HAdV-B which includes viruses from mixed host origin [14], originated from gorillas and have switched to humans and to chimpanzees during two different host switch events [18]. Serological surveys have found that anti-AdV antibodies were prevalent in 96 % of mountain gorillas, suggesting that AdVs are circulating among these animals [19]. In addition, Hoppe et al. recently reported high prevalence of AdV in wild apes including gorillas (45−100 %) [18]. Because AdVs are shed in the feces and saliva of infected animals [13], these viruses could possibly be transmitted among host animals via the fecal–oral route and inhalation of aerosols [20].

Comprehensive studies are still needed to clarify the origin and the diversity of adenoviruses spread in human and non-human primate populations.

Thus, to fill the gap, understanding the evolution pattern of AdVs spread in non-human primates and in people frequently coming in contact with these animals is critical.

In this study, we investigated AdV infection in two habituated western lowland gorilla groups in Moukalaba-Doudou national park (MDNP). In addition, we assessed AdV infection in the local people living around the national park to evaluate potential zoonotic transmissions.

## Materials and methods

### Sample collection and preparation

**Fig. 1:**
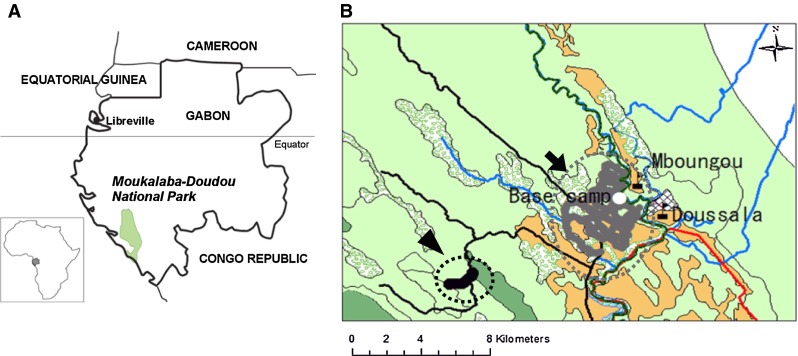
Location features of the sampling area. **a** Map of Gabon, showing Moukalaba-Doudou National Park [21]. **b** The sampling area in the MDNP (*blue line*: rivers; *black line*: roads; *red line*: hunting area limitation; *green line with black strips*: national park limitation; *dark green*: primary forest; *olive green*: secondary forest; *brown*: savanna; *spotted green*: swamp; *black circle*: sampling points of G8 pointed by *an arrow*; *gray circle* pointed by an *arrowhead*: sampling points of GG; *white circle*: base camp; *black rectangle with a black flag*: village; *white squares*: habitations) (Color figure online)

The study site MDNP is located in the south-western part of Gabon (Fig. 1). MDNP has been reported to have a high gorilla density (more than three gorillas per square kilometer) [21], and the absence of hunting pressure from local villagers makes it a major habitat for western lowland gorillas in central Africa. From December 2010 to November 2011, during tracking, we collected 112 fresh fecal samples from 2 wild gorilla groups, which were named Group Gentil (GG) and Group 8 (G8). GG and G8 had been habituated to human observers since 2003 [21] and 2011, respectively. During the study period, GG consisted of 20−21 individuals, including 1 adult (expected age ≥13 years old) male, 6 adult (≥10 years old) females, 10 young (4–6 years old) males, and 3 young females, and all members were individually identified. In contrast, G8 was estimated to consist of 8–12 individuals, including 1 adult male, 2 adult females, and 5−8 young males and females. GG was mainly sampled near the village Doussala, in the ancient plantations, where the forest has been formerly used in various crop fields, while G8 was found far from the village in the primary forest (Fig. 1). In addition to the gorilla samples, 20 fecal samples were collected from villagers, including trackers working for the habituation of gorillas. Upon collection, each fecal specimen was immediately placed into a tube containing 2 ml of RNAlater (Ambion, Austin, TX, USA). The tubes were kept at room temperature for at most 20 days at the field camp until the samples were transported to the laboratory in Libreville, the capital city of Gabon. At the laboratory, the tubes were stored at –20 °C until DNA extraction.

### DNA extraction and PCR

Total DNA was extracted from the sample using the QIAamp DNA Stool Mini Kit (Qiagen, Hilden, Germany) according to the manufacturer’s instructions. We used the following primer sets for nested PCR: (1) 4431-s/4428-as and 4428-s/4429-as (Supplementary Table 1), targeting the HAdV DNA polymerase (*DPOL*) gene [14] and (2) AdhexF1/AdhexR1 and AdhexF2/AdhexR2, targeting loop 1—encompassing the hypervariable region (HVR_1–6_)—of the hexon gene of mastadenoviruses [22]. PCR for the *DPOL* gene was performed in a total volume of 20 µl containing 10 µl of 2× GoTaq Green Master Mix (Promega, Madison, WI, USA), 20 pmol of each primer, and 50 ng of DNA template. The following cycling conditions, slightly modified from Wevers et al. [14], were used: 95 °C for 2 min; 35 cycles of 95 °C for 30 s, 55 °C for 1 min, and 72 °C for 1 min; and a 7-min final extension step at 72 °C. PCR amplification of the hexon gene (HVR_1–6_) was performed in a total volume of 50 µl containing 200 µM of each dNTP, 20 pmol of each primer, 1.25 U of PrimeSTAR GXL polymerase (TaKaRa, Tokyo, Japan), and 50 ng of DNA template. The cycling conditions were as follows: 98 °C for 3 min; 35 cycles of 98 °C for 10 s, 45 °C for 1 min, and 72 °C for 2 min; and a final extension of 72 °C for 7 min. For the nested reaction, 2 µl of the first PCR product was amplified as above. Amplified products were separated on 1.5 % agarose gel and purified using the QIAquick Gel Extraction Kit (Qiagen) according to the manufacturer’s instructions; the amplicons were then directly sequenced with the primers for the second PCR.

### BLAST search

BLAST searches were carried out in the NCBI database (http://blast.ncbi.nlm.nih.gov/Blast.cgi) using the determined nucleotide sequence as a query in the BLASTN program. The queries with at least 90 % identity with the deposited adenovirus gene sequences were considered for AdV species identification.

### Sequencing and phylogenetic analysis

Twenty-four of the 27 positive samples (DNA quantity ≥5 ng/µl) were subjected to direct sequencing of *DPOL* gene fragments. Six samples were selected randomly for cloning and sequencing of *DPOL* and hexon HVR_1–6_ gene fragments. The PCR products were cloned into plasmid vector pCR-Blunt II-TOPO using the Zero Blunt TOPO PCR cloning kit (Invitrogen, Carlsbad, CA, USA) according to the manufacturer’s instructions. Plasmid extraction was carried out using the Wizard Miniprep Kit (Promega), and the extracted plasmids were sequenced by Big Dye terminator cycle sequencing (Applied Biosystems, Foster City, CA, USA).

The hexon HVR_1–6_ and *DPOL* gene sequences were edited and aligned using GENETYX software version 12.0 (Genetyx Co., Tokyo, Japan) and MEGA software version 5.05 [23]. The nucleotide sequences of *DPOL* (528-bp, corresponding to the position 29,200–29,727 in the reference simian adenovirus 21*)* and 782-bp fragments of the hexon gene (corresponding to the position 18,867–19,635 in the reference simian adenovirus 21) were aligned using MUSCLE, with the default parameters for gap opening and gap extension. These alignments were used for phylogenetic analyses. Phylogenetic trees were constructed using the neighbor-joining method in MEGA 5.05 [23]. A statistical test for the phylogeny was computed by means of bootstrapping. Percentages of 100 bootstrap replicates at the node were calculated to ensure the reliability of the trees.

### Nucleotide sequence accession numbers

Preliminary names were given to candidate novel HAdVs following the method used by Wevers et al. [14]. The gorilla adenoviruses detected in this study were named as follows: Gorilla gorilla AdV B11-B23 (KM886307-KM886309, KM886311, KM886325-KM886328, KM886331-KM886335), Gorilla gorilla AdV C10-C18 (KM886310, KM886320-KM886324, KM886329), and Gorilla gorilla AdV E1 (KM886330). The sequences used as references for phylogenetic analysis are presented in Supplementary Table 2.

## Results

### Detection of AdV genes in western lowland gorillas in MDNP

To survey AdV infection in gorillas in MDNP, we collected fecal samples from two gorilla groups (GG: well-habituated group, G8: newly habituated group) and analyzed them by nested PCR targeting the *DPOL* and hexon genes. The *DPOL* and hexon genes were detected in both groups (Table 1). The overall prevalence of AdV in the gorilla population was 24.1 % (27/112): of the 86 samples from GG, 21 were positive for both genes, 4 were positive only for the *DPOL* gene, and 1 sample was positive only for the hexon gene. In contrast, only 1 of the 26 samples was positive for both tested genes in G8 (Table 1). These data suggest that AdVs are naturally circulating among gorillas in MDNP. To confirm the detected AdV species, we further determined the nucleotide sequences of the amplicons and determined the species of the detected AdVs by BLAST searches. Of the tested samples, 16 belonged to HAdV-B; 10 to HAdV-C; and 1 to HAdV-E.

**Table 1:** Detection of adenovirus *DPOL* and hexon genes in samples from gorilla groups in MDNP

Gorilla groups	No. of tested samples	No. of positive samples in PCR (%)	HAdV Species		
			No. of samples		
			B	C	E
GG	86	26 (30.2 %)	16	9	1
G8	26	1 (3.8 %)	0	1	0
Total	112	27 (24.1 %)	16	10	1

### Detection of AdV genes in local people living around the national park

The prevalence of AdVs in well-habituated gorillas (30.2 % in GG group) was higher than that of newly habituated ones (3.8 % in G8 group), raising two possibilities either the AdVs in gorillas are derived from humans during the habituation process or AdVs are ubiquitous in the environment in and around the areas of human habitation. Therefore, we screened the local people (village Doussala in Fig. 1) for AdV infection. The prevalence in the local people was 35.0 % (7/20): two samples were positive for both *DPOL* and hexon genes, and five were positive only for the hexon gene (Table 2). These results revealed that the local people including trackers were also infected with AdVs. We sequenced the detected virus genes and identified the species of AdVs: one sample was infected with a HAdV-C type, and the others harbored HAdV-D members.

**Table 2:** Adenovirus infection in humans

Sample ID	PCR DPOL	PCR hexon
H1	HAdV-C	HAdV-C
H2		HAdV-D
H3		
H4		
H5		
H6		HAdV-D
H7		
H8		HAdV-D
H9		
H10		
H11		HAdV-D
H12		HAdV-D
H13		
H14		
H15		
H16		
H17	HAdV-D	HAdV-D
H18		
H19		
H20		

### Phylogenetic analysis

HAdV-C genes were detected in both gorillas and humans in MDNP, suggesting zoonotic transmission of AdV between the human and gorilla populations. To investigate this possibility, as well as to gain insights into the genetic diversity of adenoviruses in MDNP, we performed phylogenetic analyses.

**Fig. 2:**
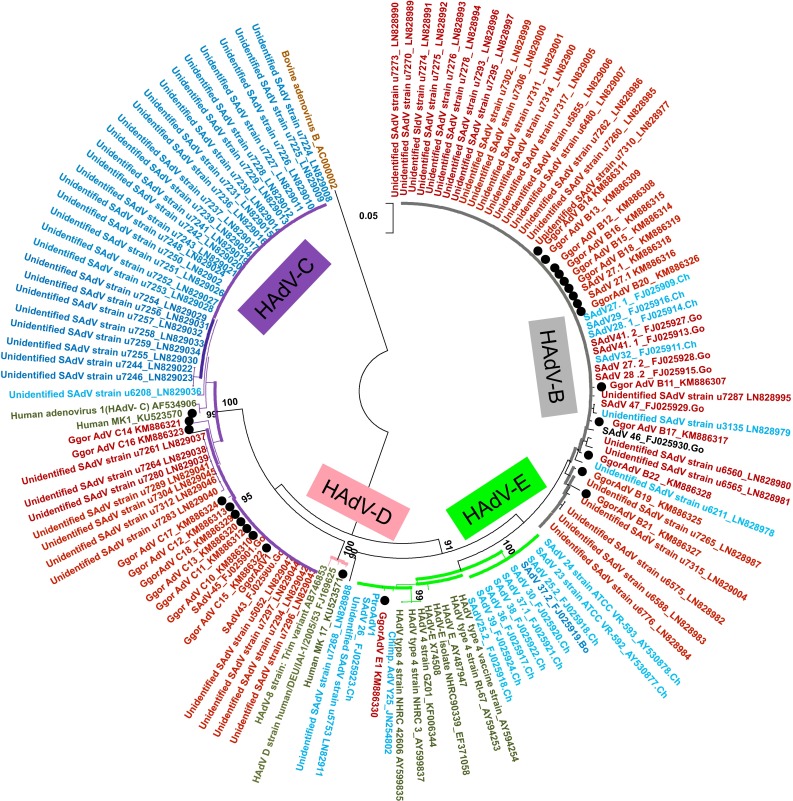
Phylogenetic tree of adenovirus (AdV) *DPOL*. The tree was constructed based on the alignment of AdV *DPOL* (539 bp) by using the neighbor-joining bootstrap-confirmed method in MEGA 5.05 software with 100 replicates. The names of simian isolates include the serotype nomenclature and the animal species of isolation (*Ch* chimpanzee, *Go* gorilla, *Bo* bonobo). Names of novel sequences obtained in this study are indicated with* black dots*. Bootstrap values <90 % are omitted. *Scale bar*, nucleotide substitutions per site

**Fig. 3:**
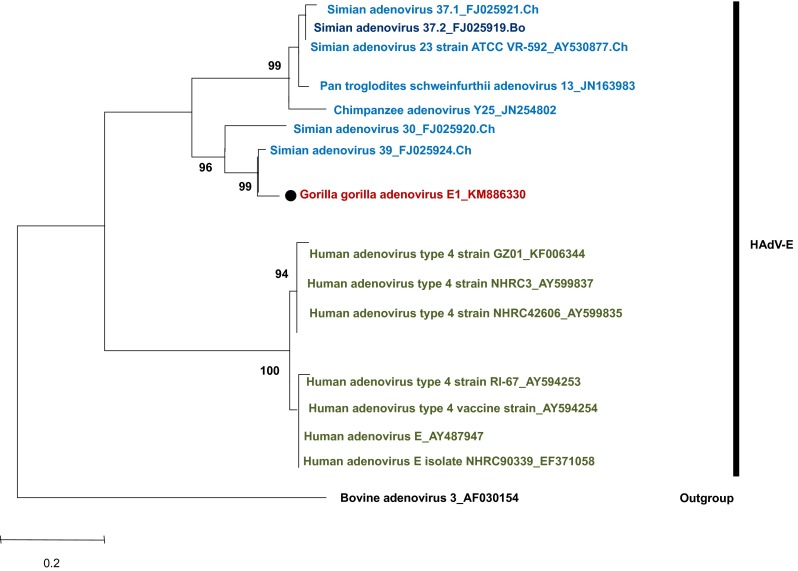
Phylogenetic tree of the adenovirus hexon gene loop 1 of HAdV-E. The tree was constructed based on the alignment of a 792-bp sequence of the hexon gene by using the neighbor-joining bootstrap-confirmed method in MEGA 5.05 software with 100 replicates. The names of simian isolates include the serotype nomenclature and the animal species of isolation (*Ch* chimpanzee, *Go* gorilla *Bo* bonobo). Names of novel sequences obtained in this study are indicated with *black dots*

In gorillas, on the tree based on the *DPOL* gene, 14 AdV genes identified in this study were divided into two groups; they clustered with SAdV-28.2, SAdV-46, SAdV-47, and gorilla AdV strains 6588 and 6575, which are representative strains of HAdV-B in gorillas, and unidentified simian adenoviruses recently described [18] (Fig. 2 and Supplementary Fig. 1). Nine AdV genes were clustered with simian AdV-45 and simian AdV-43, which are representative strains of HAdV-C in gorilla and new unidentified simian adenoviruses [18] (Fig. 2 and Supplementary Fig. 1). In contrast, one AdV gene clustered with SAdV-26 and chimpanzee AdV strain Y25, which are chimpanzee-specific strains belonging to HAdV-E (Fig. 2 and Supplementary Fig. 1). On the hexon gene-based trees, five HAdV-B (Supplementary Fig. 2a) and HAdV-E (Fig. 3) strains were identified among those isolated from gorillas. HADV-E is divided into four groups (Fig. 3): two groups of human origin and two of simian origin. The HAdV-E detected in gorillas in this study belonged to the *H mastadenovirus E* of simian origin (Fig. 3).

In the case of humans, the tree based on the *DPOL* gene showed one AdV gene clustered with HAdV-1 (HAdV-C), which is genetically different from the strains detected in gorillas (Fig. 2 and Supplementary Fig. 2b), and one clustered with the human AdV type 44 and human AdV type 47, which belong to the HAdV-D (Supplementary Fig. 1). The HAdV-D seems to be exclusively limited to the human population as reported earlier [18].

## Discussion

In this study, we detected several species of AdVs in western lowland gorillas in MDNP as well as in local people residing nearby. Interestingly, the positive rate in the well-habituated group (30.2 %) was higher than that of the newly habituated group (3.8 %). In addition, members of HAdV-C were detected in both gorillas and humans. However, the phylogenetic analyses revealed that the AdVs detected from gorillas are genetically distinct from those from local people living around the national park. Therefore, gorilla viruses and human viruses may have been separately circulating in each population in this region, and transmission between human and animals does not seem to happen easily in either direction, although we cannot exclude the possibility that we just missed zoonotically transmitted AdVs in this study. The difference in the prevalence between groups GG and G8 may be attributed to the quality of samples, because samples from GG might have been fresher than the ones from G8; GG was sampled while following animals, but G8 was sampled on trails, sometimes without observing the animals. In contrast, AdVs were reported to be transmitted between humans and non-human primates, indicating that AdVs have zoonotic potential [15, 18], despite the belief that AdVs have co-evolved with their hosts and are usually not transmitted to other species.

Adenovirus infections have been reported in high prevalence in wild gorillas’ populations as well as in other great apes [15, 17, 18]. In this study, the overall prevalence of AdV infection in gorillas was 24.1 %, which is lower than the previously reported figure of 44.9 % in free-ranging gorillas in Congo Republic [17] or of 48 % in free-ranging gorillas in Loango National Park (Gabon) [18]. These differences might be due to the quality of the samples and/or sensitivity of the PCR. In addition, the PCR systems used in this study targeted the conserved DNA polymerase gene of mastadenovirus or the hypervariable region of the hexon gene, but in some samples, only one of the two genes was amplified. This shows that our PCR system might not be able to amplify all gene variants or that the samples could have been partially degraded [18]. Alternatively, the DNA amount of AdV in the gorillas included in this study was lower than detection limit. Further systematic studies are needed to assess these possibilities.

We detected members of three species: HAdV-B, HAdV-C, and HAdV-E in western lowland gorillas in MDNP; these AdV species have been reported earlier [15–18] in western lowland gorillas as well as in other gorilla sub-species in sub-Saharan Africa. The gorilla adenoviruses of this study mainly belong to the HAdV-B (59 %). This confirms the gorilla as the major host of HAdV-B in sub-Saharan Africa. Based on the hexon tree (Supplementary Fig. 2a), the new virus named Gorilla gorilla adenovirus B19, together with the *Human mastadenovirus B* isolates 6560 and 6674 constitutes a single clade. The pattern observed within the species *Human mastadenovirus C* (Supplementary Fig. 1) is compatible with the host-pathogen divergence as previously reported [13, 15, 18]. All the lineages in HAdV-C are host specific [18]. The only member of HAdV-E detected in this study clusters with chimpanzee strains (Fig. 3). This finding supports previous report describing the non-human primate AdVs members of the HAdV-E to originate from chimpanzees [18]. We can suspect the Gorilla gorilla adenovirus E1 of this study to be the result of chimpanzee-to-gorilla transmission, as chimpanzees and gorillas are living sympatrically in MNDP. Broader screening would clarify the evolution of viruses belonging to HAdV-E.

On the other hand, the adenoviruses detected in the human population around MDNP are mainly members of the HAdV-D (85.71 %) which confirms that the species HAdV-D originated in humans [18] and so far has been exclusively human specific. Four different serotypes were detected in this study, highlighting the diversity of adenoviruses circulating in the target human population. Further systematic studies should clarify the circulation of AdVs in human population.

Taken together, our results show that AdVs are naturally present among gorillas and humans in MDNP in Gabon. Although there is no evidence of zoonotic transmission of AdVs in this region, our data show the feasibility of monitoring viral agents in wild habituated gorillas [24] and in local people living nearby for the safe management of wild gorilla populations and human health, as well as for understanding the evolution of virus. Since the zoonotic transmission of adenovirus already occurred during hominin evolution, assessing the zoonotic transmission of that virus in the context of habituation sites such as MNDP is recommended.

## Electronic supplementary material

Below is the link to the electronic supplementary material.

Supplementary material 1 (DOC 1146 kb)
