# Supplementary material for: Molecular epidemiological study of adenovirus infecting western lowland gorillas and humans in and around Moukalaba-Doudou National Park (Gabon)
Source: Virus Genes. 2016 Jun 11;52(5):671–8. doi: 10.1007/s11262-016-1360-8 (PMC5002280; doi:10.1007/s11262-016-1360-8)
Supplement: Supplementary file 1 — Supplementary material 1 (DOC 1146 kb) [file 11262_2016_1360_MOESM1_ESM.doc]

| **Primer set abbreviation** |  | **Targeted gene** | | **Name of primer** | **Sequence 5’-3’** | **PCR length** |
| --- | --- | --- | --- | --- | --- | --- |
| *DPOL*-cons |  | | *DPOL*  1^st^ round  2^nd^ round | 4431-s | GTnTwyGAyAThTGyGGhATGTAyGC | 956 |
|  |  | |  | 4428-as | GAGGCTGTCCGTrTC(n/I)CCGTA^#^ |  |
|  |  | |  | 4428-s | CGGACGCCTCTGyTGGAC(n/I)AA | 650 |
|  |  | |  | 4429-as | GGCCAGCACrAA(n/I)GArGC |  |
| HVR(_1-6_) |  | | Hexon | AdhexF1 | TICTTTGACATICGIGGIGTICTIGA | 850 |
|  |  | | 1^st^ round | AdhexR1 | CTGTCIACIGCCTGRTTCCACA |  |
|  |  | |  | AdhexF2 | GGYCCYAGYTTYAARCCCTAYTC | 774 |
|  |  | | 2^nd^ round | AdhexR2 | GGTTCTGTCICCCAGAGARTCIAGCA |  |

**Supplementary table 1. Primers and probe sequences for amplification of *DPOL***

**and hexon genes**

s=sense, as=antisense #I= inosine, n= any base (not a gap)

| **Supplementary Table 2: Adenoviruses, accession number and host** | | | | | |
| --- | --- | --- | --- | --- | --- |
| **Adenovirus** | **Abbreviation** | **GenBank accession number/reference** | **Host** | **Wild** | **Captive^a^** |
| **HAdV-B of this study** |  |  |  |  |  |
| Gor. gorilla adenovirus B11 | Ggor AdV B11 | **KM886307** | Gorilla | + |  |
| Gor. gorilla adenovirus B12 | Ggor AdV B12 | **KM886308** | Gorilla | + |  |
| Gor. gorilla adenovirus B13 | Ggor AdV B13 | **KM886309** | Gorilla | + |  |
| Gor. gorilla adenovirus B14 | Ggor AdV B14 | **KM886311** | Gorilla | + |  |
| Gor. gorilla adenovirus B15 | Ggor AdV B15 | **KM886314** | Gorilla | + |  |
| Gor. gorilla adenovirus B16 | Ggor AdV B16 | **KM886315** | Gorilla | + |  |
| Gor. gorilla adenovirus B17 | Ggor AdV B17 | **KM886317** | Gorilla | + |  |
| Gor. gorilla adenovirus B18 | Ggor AdV B18 | **KM886319** | Gorilla | + |  |
| Gor. gorilla adenovirus B19 | Ggor AdV B19 | **KM886325** | Gorilla | + |  |
| Gor. gorilla adenovirus B20 | Ggor AdV B20 | **KM886326** | Gorilla | + |  |
| Gor. gorilla adenovirus B21 | Ggor AdV B21 | **KM886327** | Gorilla | + |  |
| Gor. gorilla adenovirus B22 | Ggor AdV B22 | **KM886328** | Gorilla | + |  |
| **HAdV-C of this study** |  |  |  |  |  |
| Gor. gorilla adenovirus C10 | Ggor AdV C10 | **KM886310** | Gorilla | + |  |
| Gor. gorilla adenovirus C11 | Ggor AdV C11 | **KM886312** | Gorilla | + |  |
| Gor. gorilla adenovirus C12 | Ggor AdV C12 | **KM886313** | Gorilla | + |  |
| Gor. gorilla adenovirus C13 | Ggor AdV C13 | **KM886320** | Gorilla | + |  |
| Gor. gorilla adenovirus C14 | Ggor AdV C14 | **KM886321** | Gorilla | + |  |
| Gor. gorilla adenovirus C15 | Ggor AdV C15 | **KM886322** | Gorilla | + |  |
| Gor. gorilla adenovirus C16 | Ggor AdV C16 | **KM886323** | Gorilla | + |  |
| Gor. gorilla adenovirus C17 | Ggor AdV C17 | **KM886324** | Gorilla | + |  |
| Gor. gorilla adenovirus C18 | Ggor AdV C18 | **KM886329** | Gorilla | + |  |
|  |  |  |  | + |  |
| **HAdV-E of this study** |  |  |  |  |  |
| Gor. gorilla adenovirus E1 | Ggor AdV E1 | **KM886330** | Gorilla | + |  |
| **Reference sequences used for phylogeny** |  |  |  |  |  |
|  |  |  |  |  |  |
|  |  |  |  |  |  |
| Gorilla gorilla adenovirus 1 | Ggor AdV1 | **Wevers et al.,2011** | Gorilla | + |  |
| 6588 Gor. gor. adenovirus | 6588 Ggor AdV | **Wevers et al.,2011** | Gorilla | + |  |
| 6575 Gor. gor. adenovirus  Human adenovirus type 18 | 6575 Ggor AdV  HAdV-A-18 | **Wevers et al.,2011**  **GU191010** | Gorilla | + |  |
| Human adenovirus type 31 | HAdV-A-31 | **AM749299** |  |  |  |
| Human adenovirus type 1 | HAdV-C | **AF534906** |  |  |  |
| Human adenovirus D-8 | HAdV D-8 | **AB448767** |  |  |  |
| Human adenovirus D-53 | HAdV D-53 | **FJ169625** |  |  |  |
| Human_adenovirus_26_:_BP-2_ |  | **AB330107** |  |  |  |
| Human_adenovirus_62_isolate_Aids25/Manchester/1993_ |  | **JN162671** |  |  |  |
| Human_adenovirus_69 |  | **JN226748** |  |  |  |
| Human_adenovirus_29 |  | **JN226754** |  |  |  |
| Human_adenovirus_54 |  | **NC012959** |  |  |  |
| Human_adenovirus_9 |  | **AJ854486** |  |  |  |
| Human adenovirus 11 |  | **AB330092** |  |  |  |
| Human adenovirus 55  Human adenovirus 35  Human adenovirus 21  Human adenovirus 14 |  | **KF911353**  **AB330116**  **AB330102**  **AB330095** |  |  |  |
| Human adenovirus 34  Human adenovirus 50  Human adenovirus 3 |  | **AB330115**  **AB33013**  **AB330084** |  |  |  |
| Human adenovirus 7 |  | **AB330088** |  |  |  |
| Human adenovirus 16  Human adenovirus 2  Human adenovirus 5  Human adenovirus 6  Human adenovirus 57  Human adenovirus 12  Human adenovirus 18 |  | **AB330097**  **AB330083**  **AB330086**  **AB330087**  **KF835458**  **X73487**  **GU191019** |  |  |  |
| Human_adenovirus_10 |  | **AB330091** |  |  |  |
| Human_adenovirus_15 |  | **AB562586** |  |  |  |
| Human_adenovirus_13 |  | **JN226747** |  |  |  |
| Human_adenovirus_17 |  | **HQ910407** |  |  |  |
| Human_adenovirus_19_ |  | **JQ326209** |  |  |  |
| Human_adenovirus_20_ |  | **JN226749** |  |  |  |
| Human_adenovirus_22_ |  | **FJ619037** |  |  |  |
| Human_adenovirus_23_ |  | **KF279629** |  |  |  |
| Human_adenovirus_24_ |  | **JN226751** |  |  |  |
| Human_adenovirus_25_ |  | **JN226752** |  |  |  |
| Human_adenovirus_27_ |  | **JN226753** |  |  |  |
| Human_adenovirus_28_ |  | **FJ824826** |  |  |  |
| Human_adenovirus_30_ |  | **JN226755** |  |  |  |
| Human_adenovirus_32_ |  | **JN226756** |  |  |  |
| Human_adenovirus_33_ |  | **JN226758** |  |  |  |
| Human_adenovirus_36_ |  | **GQ384080** |  |  |  |
| Human_adenovirus_37_ |  | **AB448778** |  |  |  |
| Human_adenovirus_38_ |  | **JN226759** |  |  |  |
| Human_adenovirus_39_ |  | **JN226760** |  |  |  |
| Human_adenovirus_42 |  | **JN226761** |  |  |  |
| Human_adenovirus_43 |  | **JN226762** |  |  |  |
| Human_adenovirus_44 |  | **JN226763** |  |  |  |
| Human_adenovirus_45 |  | **JN226764** |  |  |  |
| Human adenovirus type 4^c^ | HAdV-4 | **AY594253** |  |  |  |
| Human adenovirus type 4^d^ | HAdV-4 | **AY594254** |  |  |  |
| Human adenovirus type 4^e^ | HAdV-4 | **AY599835** |  |  |  |
| Human adenovirus -E | HAdV-E | **X74508** |  |  |  |
| Human adenovirus-E | HAdV-E | **AY487947** |  |  |  |
| Human adenovirus-E | HAdV-E | **EF371058** |  |  |  |
| Human adenovirus type 4^f^ | HAdV-4 | **AY599837** |  |  |  |
| Human adenovirus type 4^g^ | HAdV-4 | **KF006344** |  |  |  |
| Human adenovirus F-40 | HAdV F-40 | **NC_001454** |  |  |  |
| Human adenovirus F-41 | HAdV-41 | **DQ 315364** |  |  |  |
| Human adenovirus G- 52 | HAdV G-52 | **DQ 923122** |  |  |  |
| Human mastadenovirus B isolate 6674 |  | **KM659157** |  |  |  |
| Human mastadenovirus B isolate 6560 |  | **KM659138** |  |  |  |
| Simian adenovirus 1 | SAdV-1 | **AY771780** | OWM^b^ |  |  |
| Simian adenovirus 3 | SAdV-3 | **AY598782.1** | OWM^b^ |  |  |
| Simian adenovirus 28.2 | SAdV -28.2 | **FJ025915** | Gorilla |  | + |
| Simian adenovirus 46 | SAdV-46 | **FJ025930** | Gorilla |  | + |
| Simian adenovirus 45 | SAdV-45 | **FJ025901** | Gorilla |  | + |
| Simian adenovirus 48 | SAdV-48 | **HQ241818.1** | OWM^b^ |  | + |
| Simian adenovirus 24 | SAdV-24 | **AY530878.1** | Chimpanzee |  | + |
| Simian adenovirus 37.2 | SAdV-37.2 | **FJ025919** | Bonobo |  | + |
| Simian adenovirus 38 | SAdV-38 | **FJ025922** | Chimpanzee |  | + |
| Simian adenovirus 30 | SAdV-30 | **FJ025920** | Chimpanzee |  | + |
| Bovine adenovirus B | BAdV-B-3 | **AC_000002** |  |  |  |
| Unidentified simian adenovirus strain u5753 |  | **LN829111** | Chimpanzee | + |  |
| Unidentified simian adenovirus strain u7312 |  | **LN829046** | Gorilla | + |  |
| Unidentified simian adenovirus strain u7304 |  | **LN829045** | Gorilla | + |  |
| Unidentified simian adenovirus strain u7289 |  | **LN829041** | Gorilla | + |  |
| Unidentified simian adenovirus strain u7283 |  | **LN829040** | Gorilla | + |  |
| Unidentified simian adenovirus strain u7280 |  | **LN829039** | Gorilla | + |  |
| Unidentified simian adenovirus strain u7264 |  | **LN829038** | Gorilla | + |  |
| Unidentified simian adenovirus strain u7261 |  | **LN829037** | Gorilla | + |  |
| Unidentified simian adenovirus strain u6208 |  | **LN829036** | Chimpanzee | + |  |
| Unidentified simian adenovirus strain u7259 |  | **LN829034** | Bonobo | + |  |
| Unidentified simian adenovirus strain u7258 |  | **LN829033** | Bonobo | + |  |
| Unidentified simian adenovirus strain u7257 |  | **LN829032** | Bonobo | + |  |
| Unidentified simian adenovirus strain u7256 |  | **LN829031** | Bonobo | + |  |
| Unidentified simian adenovirus strain u7255 |  | **LN829030** | Bonobo | + |  |
| Unidentified simian adenovirus strain u7254 |  | **LN829029** | Bonobo | + |  |
| Unidentified simian adenovirus strain u7253 |  | **LN829028** | Bonobo | + |  |
| Unidentified simian adenovirus strain u7252 |  | **LN829027** | Bonobo | + |  |
| Unidentified simian adenovirus strain u7251 |  | **LN829026** | Bonobo | + |  |
| Unidentified simian adenovirus strain u7250 |  | **LN829025** | Bonobo | + |  |
| Unidentified simian adenovirus strain u7248 |  | **LN829024** | Bonobo | + |  |
| Unidentified simian adenovirus strain u7246 |  | **LN829023** | Bonobo | + |  |
| Unidentified simian adenovirus strain u7244 |  | **LN829022** | Bonobo | + |  |
| Unidentified simian adenovirus strain u7243 |  | **LN829021** | Bonobo | + |  |
| Unidentified simian adenovirus strain u7242 |  | **LN829020** | Bonobo | + |  |
| Unidentified simian adenovirus strain u7241 |  | **LN829019** | Bonobo | + |  |
| Unidentified simian adenovirus strain u7239 |  | **LN829018** | Bonobo | + |  |
| Unidentified simian adenovirus strain u7237 |  | **LN829017** | Bonobo | + |  |
| Unidentified simian adenovirus strain u7236 |  | **LN829016** | Bonobo | + |  |
| Unidentified simian adenovirus strain u7231 |  | **LN829015** | Bonobo | + |  |
| Unidentified simian adenovirus strain u7230 |  | **LN829014** | Bonobo | + |  |
| Unidentified simian adenovirus strain u7229 |  | **LN829013** | Bonobo | + |  |
| Unidentified simian adenovirus strain u7228 |  | **LN829012** | Bonobo | + |  |
| Unidentified simian adenovirus strain u7227 |  | **LN829011** | Bonobo | + |  |
| Unidentified simian adenovirus strain u7226 |  | **LN829010** | Bonobo | + |  |
| Unidentified simian adenovirus strain u7225 |  | **LN829009** | Bonobo | + |  |
| Unidentified simian adenovirus strain u7224 |  | **LN829008** | Bonobo | + |  |
| Unidentified simian adenovirus strain u7315 |  | **LN829004** | Gorilla | + |  |
| Unidentified simian adenovirus strain u7287 |  | **LN828995** | Gorilla | + |  |
| Unidentified simian adenovirus strain u7273 |  | **LN828990** | Gorilla | + |  |
| Unidentified simian adenovirus strain u7268 |  | **LN828988** | Gorilla | + |  |
| Unidentified simian adenovirus strain u7265 |  | **LN828987** | Gorilla | + |  |
| Unidentified simian adenovirus strain u6776 |  | **LN828984** | Gorilla | + |  |
| Unidentified simian adenovirus strain u6588 |  | **LN828983** | Gorilla | + |  |
| Unidentified simian adenovirus strain u6575 |  | **LN828982** | Gorilla | + |  |
| Unidentified simian adenovirus strain u6565 |  | **LN828981** | Gorilla | + |  |
| Unidentified simian adenovirus strain u6560 |  | **LN828980** | Gorilla | + |  |
| Unidentified simian adenovirus strain u3135 |  | **LN828979** | Chimpanzee | + |  |
| Unidentified simian adenovirus strain u6211 |  | **LN828978** | Chimpanzee | + |  |
| Unidentified simian adenovirus strain u5052 |  | **LN829047** | Gorilla | + |  |
| Unidentified simian adenovirus strain u7297 |  | **LN829044** | Gorilla | + |  |
| Unidentified simian adenovirus strain u7296 |  | **LN829043** | Gorilla | + |  |
| Unidentified simian adenovirus strain u7294 |  | **LN829042** | Gorilla | + |  |
| Unidentified simian adenovirus strain u6480 |  | **LN829007** | Gorilla | + |  |
| Unidentified simian adenovirus strain u5855 |  | **LN829006** | Gorilla | + |  |
| Unidentified simian adenovirus strain u7317 |  | **LN829005** | Gorilla | + |  |
| Unidentified simian adenovirus strain u7314 |  | **LN829003** | Gorilla | + |  |
| Unidentified simian adenovirus strain u7311 |  | **LN829001** | Gorilla | + |  |
| Unidentified simian adenovirus strain u7306 |  | **LN829000** | Gorilla | + |  |
| Unidentified simian adenovirus strain u7302 |  | **LN828999** | Gorilla | + |  |
| Unidentified simian adenovirus strain u7295 |  | **LN828997** | Gorilla | + |  |
| Unidentified simian adenovirus strain u7293 |  | **LN828996** | Gorilla | + |  |
| Unidentified simian adenovirus strain u7278 |  | **LN828994** | Gorilla | + |  |
| Unidentified simian adenovirus strain u7276 |  | **LN828993** | Gorilla | + |  |
| Unidentified simian adenovirus strain u7275 |  | **LN828992** | Gorilla | + |  |
| Unidentified simian adenovirus strain u7274 |  | **LN828991** | Gorilla | + |  |
| Unidentified simian adenovirus strain u7270 |  | **LN828989** | Gorilla | + |  |
| Unidentified simian adenovirus strain u7262 |  | **LN828986** | Gorilla | + |  |
| Unidentified simian adenovirus strain u7260 |  | **LN828985** | Gorilla | + |  |
| Unidentified simian adenovirus strain u7310 |  | **LN828977** | Gorilla | + |  |

aCaptive: zoo animals bOWM: Old world monkey c: strain RI-67, d: vaccine strain, e: strain NHRC 42606, f: strain NHRC 3, g: strain GZ01

**Supplementary figure 1: Phylogenetic tree of the partial sequence of *DPOL***

The tree was constructed based on the alignment of AdV *DPOL* (539 bp) by using the neighbor-joining bootstrap-confirmed method in MEGA 5.05 software with 100 replicates. The names of simian isolates include the serotype nomenclature and the animal species of isolation (Ch: chimpanzee, Go: gorilla, Bo: bonobo). Names of novel sequences obtained in this study are indicated with black dots. Bootstrap values less than 90% are omitted. Scale bar, nucleotide substitutions per site.

**Supplementary figure 2: Phylogenetic tree of the partial sequence of the hexon gene**

The tree was constructed based on the alignment of a 792-bp sequence of the hexon gene by using the neighbor-joining bootstrap-confirmed method in MEGA 5.05 software with 100 replicates. The names of simian isolates include the serotype nomenclature and the animal species of isolation (Ch: chimpanzee, Go: gorilla Bo: bonobo). Names of novel sequences obtained in this study are indicated with black dots.

**Supplementary figure 3: Phylogenetic tree of partial hexon of HAdV-D**

The tree was constructed based on the alignment of a 792-bp sequence of hexon gene by using the neighbor-joining bootstrap-confirmed method in MEGA 5.05 software with 100 replicates. The names of simian isolates include the serotype nomenclature and the animal species of isolation (Ch: chimpanzee, Go: gorilla Bo: bonobo). Names of novel sequences obtained in this study are indicated with black dots.
